# Study on the Association Network of Tuberculosis Lesions in Adult Extrapulmonary Tuberculosis in China: A Large-Scale Multicenter Observational Study

**DOI:** 10.1155/cjid/4944872

**Published:** 2025-05-16

**Authors:** Jiajia Yu, Yunqing Chang, Chen Liang, Shengsheng Liu, Liang Li, Jian Du, Youcai Li, Hongyan Chen, Jianxiong Liu, Jinshan Ma, Mingwu Li, Jingmin Qin, Wei Shu, Peilan Zong, Yi Zhang, Xiaofeng Yan, Zhiyi Yang, Yongkang Dong, Zaoxian Mei, Qunyi Deng, Pu Wang, Wenge Han, Meiying Wu, Ling Chen, Xinguo Zhao, Lei Tan, Fujian Li, Chao Zheng, Hongwei Liu, Xinjie Li, Ertai A, Yingrong Du, Fenglin Liu, Wenyu Cui, Song Yang, Xiaohong Chen, Quanhong Wang, Junfeng Han, Qingyao Xie, Yanmei Feng, Wenyu Liu, Peijun Tang, Jianyong Zhang, Jian Zheng, Dawei Chen, Xiangyang Yao, Tong Ren, Yang Li, Yuanyuan Li, Lei Wu, Qiang Song, Jian Zhang, Mei Yang, Yuanyuan Liu, Shuliang Guo, Kun Yan, Xinghua Shen, Dan Lei, Yangli Zhang, Shenjie Tang, Wanli Kang

**Affiliations:** ^1^Department of Infectious Diseases and Clinical Microbiology, Beijing Chao-Yang Hospital, Capital Medical University, Beijing, China; ^2^Beijing Chest Hospital, Capital Medical University, Beijing Tuberculosis and Thoracic Tumor Research Institute, Beijing, China; ^3^Department of Infectious Diseases, Shanxi Bethune Hospital, Shanxi Academy of Medical Sciences, Tongji Shanxi Hospital, Third Hospital of Shanxi Medical University, Taiyuan, Shanxi Province, China; ^4^Department of Infectious Diseases, Beijing Tsinghua Changgung Hospital, School of Clinical Medicine, Tsinghua University, Beijing, China; ^5^Department of Tuberculosis, Anhui Chest Hospital/Anhui Provincial Institute for Tuberculosis Prevention and Treatment, Hefei, China; ^6^Shenyang Chest Hospital, No. 11 Beihai Street, Dadong District, Shenyang, China; ^7^Guangzhou Chest Hospital, No. 62, Heng Zhi Gang Road, Yuexiu District, Guangzhou, Guangdong, China; ^8^Chest Hospital of Xinjiang, No. 106 Yan'An Road, Tianshan District, Urumqi, Xinjiang, China; ^9^The Third People's Hospital of Kunming, No. 319 Wujing Road, Kunming City, Yunnan Province, China; ^10^Shandong Provincial Chest Hospital, No. 12 Lieshishandong Road, Licheng District, Jinan, Shandong, China; ^11^Jiangxi Chest (Third People) Hospital, No. 346 Dieshan Road, Donghu District, Nanchang City, Jiangxi Province, China; ^12^Chang Chun Infectious Diseases Hospital, No. 2699, Sandao Section, Changji South Line, Erdao District, Changchun City, Jilin Province, China; ^13^Chongqing Public Health Medical Center, No. 109, Baoyu Road, Geleshan Town, Shapingba District, Chongqing, China; ^14^Fuzhou Pulmonary Hospital of Fujian, No. 2, Lakeside, Cangshan District, Fuzhou, China; ^15^Taiyuan Fourth People's Hospital, Number 231, Xikuang Street, WanBailin District, Taiyuan City, Shanxi Province, China; ^16^Tianjin Haihe Hospital, Number 890, Shuanggang Zhen Jingu Road, Jinnan District, Tianjin, China; ^17^Third People's Hospital of Shenzhen, 29 Bulan Road, District Longgang, Shenzhen, China; ^18^The First Affiliated Hospital of Chongqing Medical University, No. 1 Youyi Road, Yuzhong District, Chongqing, China; ^19^Weifang No.2 People's Hospital, No. 7th Yuanxiao Street Kuiwen District, Weifang, China; ^20^The Fifth People's Hospital of Suzhou, No. 10 Guangqian Road, Suzhou City, Jiangsu Province, China; ^21^Affiliated Hospital of Zunyi Medical College, No. 149 Delian Road, Zunyi, Guizhou, China; ^22^The Fifth People's Hospital of Wuxi, No.1215, GuangRui Road, Wuxi, China; ^23^TB Hospital of Siping City, No. 10 Dongshan Road, Tiedong District, Siping City, Jilin Province, China; ^24^Baoding Hospital for Infectious Disease, No. 608 Dongfeng East Road, Lianchi District, Baoding City, Hebei Province, China; ^25^The First Affiliated of XiaMen University, Zhenhai Roud, Siming District, Xiamen City, Fujian Province, China

## Abstract

**Background:** Extrapulmonary tuberculosis (EPTB) is a significant health problem which can lead to severe morbidity and mortality. In clinical practice, EPTB can have a variety of nonspecific clinical manifestations and can be concurrent with other types of EPTB. As information pertaining to concurrent EPTB is scarce, research efforts are needed to find concurrent EPTB types and to explore the association networks and rules of concurrent EPTB.

**Materials and Methods:** An observational multicenter study was carried out at 21 hospitals from 15 provinces in China from Jan 1, 2011, to Dec 31, 2017. All the adult EPTB inpatients (≥ 15 years) were included. Multivariable logistic regression analysis was used to examine the associations of gender and age group for concurrent EPTB. The association network and rules for concurrent EPTB were analyzed by the Apriori algorithm.

**Results:** A total of 75,993 adult EPTB inpatients (not including EPTB concurrent with PTB) were included. The ratio of male:female was 1.32. The most common types of EPTB lesions were tuberculous pleurisy (46.47%). In the fully adjusted multivariable logistic regression models, it was found that female EPTB patients (aOR = 1.129, 95% CI: 1.081–1.178) were more likely to have concurrent EPTB. As age increased, the risk of concurrent EPTB decreased (aOR < 1, *p* value for trend < 0.001). The association network graph showed that almost all the EPTB diseases may be concurrent with other types of EPTB. Ureteric tuberculosis and sacral tuberculosis diseases existed mainly in concurrence with other types of EPTB (about 80%). Tuberculous pleurisy and tuberculous lymphadenitis of the neck could be concurrent with more than 60 other types of EPTB disease. The most common concurrent EPTB types were tuberculous peritonitis concurrent with tuberculous pleurisy (1.64%). Sacral tuberculosis concurrentwith lumbar vertebra tuberculosis had the highest confidence value (68.56%). The strongest association rule was found for vesical tuberculosis concurrent with ureteric tuberculosis (lift = 166.18) and ureteric tuberculosis concurrent with vesical tuberculosis (lift = 166.18).

**Conclusion:** The present study revealed the occurrence of concurrent EPTB types and analyzed the association network and rules among adult EPTB for the first time in a large sample population. Clinicians should be alert to the incidence of concurrent EPTB and that these patients require administration of customized treatment regimens in order to achieve the best outcomes.

## 1. Introduction

Tuberculosis (TB) is an infectious disease caused by the bacillus *Mycobacterium tuberculosis* (Mtb) and is a major global health problem. According to the World Health Organization (WHO), the estimated global incidence of TB was 9.87 million cases in 2020 [1]. TB is a multisystemic disease with a protean presentation. Compared to pulmonary TB (PTB), relatively less attention has been given to extrapulmonary TB (EPTB) from public health entities, likely due to the fact that most forms of EPTB do not contribute to TB transmission [2]. In fact, 16% of reported TB cases globally in 2019 were EPTB [3]. While not usually transmissible, EPTB can cause serious morbidity and mortality [4–6]. TB is transmitted when people afflicted with PTB expel TB bacteria into the air; these airborne bacilli are subsequently inhaled by another person and settle in the lungs and grow. If the immune system does not stop TB germs from growing, the TB germs begin to multiply in the body. From there they can disseminate throughout the lymphatic or hematogenous systems and subsequently infect single or multiple extrapulmonary sites, such as the pleura, lymph nodes, and meninges [7]. The mechanisms of EPTB dissemination are complicated [8].

EPTB has a wide variety of nonspecific clinical presentations according to the location of the TB lesions. In clinical practice, EPTB concurrent with other types of EPTB (concurrent EPTB) is often observed, but little information exists about the association networks of concurrent EPTB. Concurrent EPTB cases are different and more difficult to treat than a single EPTB lesion [9]. The proportion of EPTB in TB patients varies greatly in different countries [6, 10]. In addition, studies from different countries reported an increase in the proportion of EPTB [2, 10]. A possible reason for this is that economic and medical improvement enables more and more patients with EPTB to seek treatment [11]. As information pertaining to concurrent EPTB is scarce, research efforts are needed to seek and diagnose concurrent EPTB types and explore the epidemiological characteristics and association networks that exist in concurrent EPTB. In this work, we address this gap in knowledge by conducting a large-scale multicenter observational study. The results can be used to alert clinicians to the occurrence of concurrent EPTB and to optimize treatment regimens used for such cases.

## 2. Methods

### 2.1. Study Subjects

The study was performed at 21 hospitals from 15 provinces in China, most of which were specialized TB hospitals. All the consecutive adult inpatients (≥ 15 years) with confirmed EPTB diagnoses from Jan 1, 2011, to Dec 31, 2017, were included in the study (not including cases of EPTB with concurrent PTB). TB was primarily categorized by the lesion site. The diagnosis of TB was based on WHO guidelines [12] and the TB clinical diagnosis standards issued by the Chinese Medical Association [13]. In general, TB was diagnosed using both traditional and modern methods based on clinical symptoms and physical signs together with results obtained by bacteriological methods (including sputum smear microscopy, bacterial culture, and molecular diagnostic methods), X-ray/CT findings, pathology, and treatment outcomes after a course of anti-TB chemotherapy.

### 2.2. Data Management and Statistical Analysis

Measures taken to guarantee data quality included both the implementation of a standardized study protocol and standardized training of research staff. Clinical characteristics of TB-afflicted inpatients (e.g., age, gender, and site of disease) were obtained from medical records. Descriptive statistical analysis included frequencies and proportions for categorical variables. Multivariable logistic regression analysis was used to examine the associations of gender/age groups and determinations of odds ratios were conducted for concurrent EPTB. Primary analyses were based on tests for trends, modeling the ordered age categories as linear terms. A *p* < 0.05 was the threshold for statistical significance.

Association rule analysis, a technique used to discover the relationships hidden in large databases, has been used in a variety of other fields [14, 15]. The Apriori algorithm makes it possible to apply a set of association rules to data mining. The principle of Apriori is based on two steps: the first step searches for item sets that exceed the minimum support threshold value while the second step generates association rules and filters them by selecting “confidence” item sets (based on a threshold) from item sets found in the first step [16, 17]. For the association rule of A concurrent with B, support, confidence, and lift were defined as follows: Support = P(A), Confidence = P(B|A), and Lift = P(A∩B)/[P(A) ∗ P(B)], whereby A is antecedent and B is consequent. Lift was used to evaluate the magnitude of association rules whereby a lift > 1 indicated a positive association rule and a lift ≥ 3 indicated a strong association rule. Association rules for concurrent EPTB were analyzed using the Apriori algorithm by setting the minimum support degree and the minimum confidence degree.

All data were collected in MS Office Excel datasheets (Microsoft, Redmond, WA, USA), and all analyses were conducted using SPSS software for Windows Version 23 and SPSS Modeler Version 14.1(IBM Corp, Armonk, NY, USA). The association network graph was made by UCINET 6.212 (Analytic Technologies, Lexington, KY, USA).

### 2.3. Ethical Approval and Consent to Participate

This was a retrospective observational analysis of de-identified medical records. Individual informed consent was not obtained. Given that the medical information of inpatients was recorded anonymously by case history and would not bring any risk to the participants, the ethics committee of the Beijing Chest Hospital, Capital Medical University, approved this study, with a waiver of informed consent from the patients.

## 3. Results

### 3.1. EPTB Patient Characteristics

A total of 75,993 adult EPTB patients (not including cases of EPTB concurrent with PTB) were included. The characteristics for our patient cohort are shown in Table 1. The ratio of male:female was 1.32. The proportions of 15–24 years and 25–34 years of age EPTB patients were the most. A total of 80 types of EPTB diseases were found. The most common types of EPTB lesions were tuberculous pleurisy (46.47%), followed by tuberculous lymphadenitis of the neck (10.49%), and tuberculous meningitis (7.22%). Approximately 13% of EPTB patients had at least two EPTB lesions.

### 3.2. The Concurrent EPTB in Different Gender and Age Groups

**Figure 1:**
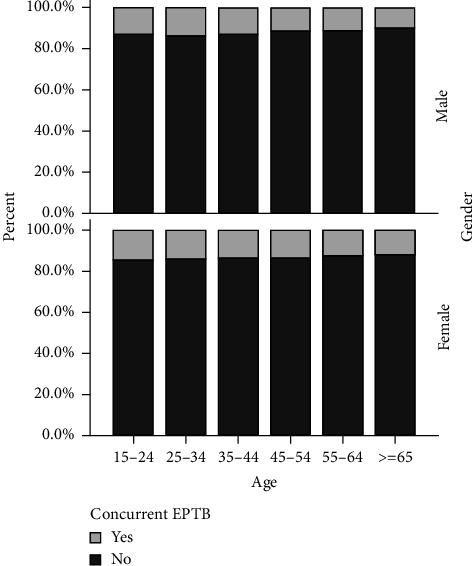
The concurrent EPTB in different gender and age groups.

 The concurrent EPTB in different gender and age groups is shown in Figure 1. Among all the age groups, the percent of concurrent EPTB in females was greater than the corresponding percent of male cases.

In the fully adjusted multivariable logistic regression models, it was found that female EPTB patients (aOR = 1.129, 95% CI: 1.081–1.178) were more likely to have concurrent EPTB. Notably, as age increased, the risk of concurrent EPTB decreased (aOR < 1, *p* value for trend < 0.001). When the ≥ 65 years age group was set as the reference, the 15–24 years group (aOR = 1.319, 95% CI: 1.225–1.421), 25–34 years group (aOR = 1.313, 95% CI: 1.219–1.414), 35–44 years group (aOR = 1.269, 95% CI: 1.172–1.375), and 45–54 years group (aOR = 1.154, 95% CI: 1.064–1.251) were more likely to have concurrent EPTB (Table 2).

### 3.3. The Association Network of Concurrent EPTB in EPTB Patients

EPTB manifested as exclusively EPTB or concurrent EPTB. It was found that many types of EPTB disease may have concurrence with other EPTB. The proportions of concurrent EPTB in different EPTB diseases were different. Some EPTB diseases existed mainly in concurrence with other types of EPTB. EPTB diseases (≥ 100 cases) with a proportion of concurrent EPTB > 50% are shown in Table 3, sorted by the proportion of concurrent EPTB. The proportion of concurrent with other types of EPTB in ureteric TB, sacral TB, and tuberculous abscess of the psoas major was more than 70%.

**Figure 2:**
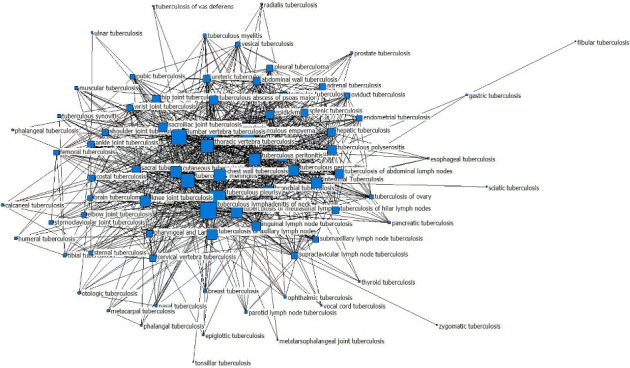
The association network of concurrent EPTB in EPTB.

The association network graph of concurrent types of EPTB in EPTB diseases is shown in Figure 2. A line connecting two types of EPTB diseases indicates that they were concurrent. For example, the connected line between tuberculous pleurisy and tuberculous peritonitis means tuberculous peritonitis is concurrent with tuberculous pleurisy. The association network graph showed that almost all the EPTB diseases may be concurrent with other types of EPTB. A bigger EPTB disease node indicates a greater number of concurrent EPTB types. For example, the node of tuberculous pleurisy was the biggest, indicating that tuberculous pleurisy has the most concurrence with other types of EPTB. EPTB diseases (≥ 100 cases) with ≥ 40 other concurrent types of EPTB disease are shown in Table 4, sorted by the concurrent types. Tuberculous pleurisy (concurrent types = 66) and tuberculous lymphadenitis of the neck (concurrent types = 64) were concurrent with more than 60 other types of EPTB disease.

The most common concurrent EPTB types (≥ 100 cases) are listed in Table 5, sorted by cases. The case numbers of tuberculous peritonitis concurrent with tuberculous pleurisy (1.64%), tuberculous pericarditis concurrent with tuberculous pleurisy (1.48%), and thoracic vertebra TB concurrent with lumbar vertebra TB (0.70%) were greater than other types of concurrent EPTB cases.

### 3.4. Association Rule Analysis of Concurrent EPTB

In order to find most of the possible association rules of concurrent EPTB, the parameters were set as follows: minimum instances were set to 100 cases, the minimum confidence degree was set to 20.00%, and lift was ≥ 1. After executing the association rule analysis, 17 association rules were obtained. The association rules are shown in Table 6 and are sorted by confidence. The first rule row(ID=1) in Table 6 was interpreted a total of 229 sacral tuberculosis cases (Instances), sacral tuberculosis accounted for 0.30% of all the EPTB cases (Support), while sacral tuberculosis concurrent with lumbar vertebra tuberculosis accounted for 68.56% of sacral tuberculosis cases (Confidence). The confidence value for ureteric TB with concurrent renal TB cases was the second highest (50.52%), followed by tuberculous pericarditis with concurrent tuberculous pleurisy (46.66%). The strongest association rule was found for vesical TB with concurrent ureteric TB (lift = 166.18), ureteric TB with concurrent vesical TB (lift = 166.18), and in TB of the hilar lymph nodes concurrent with TB of the mediastinal lymph nodes (lift = 24.82), whereby the lift value of ≥ 3 indicated that ureteric TB was positively associated with vesical TB, vesical TB was positively associated with ureteric TB, and TB of mediastinal lymph nodes was positively associated with TB of hilar lymph nodes.

## 4. Discussion

TB has many forms of extrapulmonary manifestations, affecting almost every organ of the body. It has been reported that there are significant differences in the susceptibility to EPTB in different parts of the body by age, race/ethnicity, sex, and country of origin [2, 10, 18]. The age-gender percent-bar-graph of concurrent EPTB showed that among all the age groups, the percent of concurrent EPTB in females was greater than the corresponding percent of male cases in Figure 1. But the results of 25–34 years (*p*=0.154) and 35–44 years (*p*=0.678) age groups were not statistically significant. According to the patient cohort of this study, the number of male EPTB patients was more than female with a male to female sex ratio of 1.32, which is consistent with the WHO's report that there are more cases of TB in males than females [1]. Tuberculous pleurisy is a common manifestation of EPTB and is the most common cause of pleural effusion [19]. Our data showed that tuberculous pleurisy is the predominant form of EPTB. Tuberculous pleurisy is thought to be caused by a delayed type (type IV) of hypersensitivity reaction following the release of mycobacterial antigens into the pleural space as a result of the rupture of a TB-infected subpleural focus in the lung [20].

It was found that females have a higher risk of developing EPTB through epidemiological studies conducted in industrialized countries and resource-limited countries with high burdens of TB [10, 21]. According to the data of this study, females with EPTB (aOR = 1.129, 95% CI: 1.081–1.178) were also more likely to have concurrent EPTB. Consistent with studies in the United States [10], Germany [18], Denmark [22], the European Union [2], and India [23], an association between female gender and EPTB is observed. In many studies, females tended to be more likely to have EPTB than males [24, 25]. In this study, it was found that female EPTB patients were more likely to have concurrent EPTB. An explanation for this finding remains elusive, but it suggests that female gender might play a role such as genetic and endocrine factors.

The proportions of concurrent EPTB in different EPTB diseases were different. Some EPTB diseases existed mainly in concurrence with other types of EPTB. Urogenital TB is a type of common extrapulmonary presentation of TB [26]. Ureteral TB generally does not occur alone and is normally concurrent with renal TB. It was reported that about 50% patients with renal TB have ureteric involvement [27, 28]. Our results showed that ureteral TB concurrent with other types of EPTB accounted for 78.87% of ureteral TB disease. The reason for this may be that Mtb from renal medullary lesions spread downstream with the urine to the ureters, the ureterovesical junction, and into the bladder [29–32]. Starting from primary PTB, bacillemia is responsible for bacillus distribution in parenchymatous organs, with possible colonization of the kidneys and prostate. Renal lesions are then initially bilateral, cortical, glomerular, and pericapillary, initiating hematogenous dissemination, and are concomitant with other hematogenic foci in the prostate and other organs outside the urogenital system [33]. Furthermore, involvement of the contralateral ureter occurs through retrograde ascending dissemination secondary to contracted bladder and vesicoureteral reflux [34]. Moreover, Mtb is introduced to the ureter via the bloodstream and lymphatic system, leading to ureteric TB [35–37]. Finally, the bacilli enter the periglomerular capillaries of the kidney, causing abscess formation, and gradually involve the ureter, bladder, prostate, seminal vesicle, epididymis, testes, or extra urinary organs, such as the lymph nodes, spleen, liver, and vertebrae [38, 39]. Therefore, ureteral TB often occurs simultaneously with renal TB.

Additionally, TB in the sacroiliac joint is not a rare finding [40]. It is reported that sacroiliac involvement is associated with 8%–10% of the cases of spinal TB [41]. Spinal TB usually follows hematogenous spread from the lung or genitourinary tract [42]. It is also possible that lymphatic drainage of the pleura or kidney may involve the para-aortic lymph nodes which may secondarily involve the vertebrae [43]. Usually, two or more contiguous vertebrae are involved in spinal TB, owing to hematogenous spread through one intervertebral artery feeding two adjacent vertebrae [44]. Once a vertebra has been sufficiently eroded by the process, the infection spreads gradually into the adjacent vertebrae via vascular and subligamentous routes [45]. Involvement of the sacrum is usually secondary to the lumbar spine and mainly caused by direct spread of infection [46]. Therefore, sacral TB is often accompanied by lumbar TB. The proportion of sacral TB concurrent with other TB is as high as 78.60% in this study. The psoas muscle lies in close proximity to a number of structures, including the diaphragm, pancreas, large and small intestines, kidney, ureter, spinal column, hip, and neighboring muscles (quadratus lumborum, erector spinae, and iliacus) [47–49]. Tuberculous abscess of the psoas major is more common in clinic settings [50, 51]. The psoas major abscess is not the primary lesion but often is caused by pus from the intervertebral space of lumbar or thoracic TB [50, 51]. The psoas major abscess is generally distributed from top to bottom. However, it is important to note that some psoas major abscesses may spread both upward and downward [51]. The abscess may rupture into the peritoneal cavity [52]. Therefore, tuberculous abscess of the psoas major concurrent with other TB accounts for a high proportion of EPTB cases; in this study, the proportion of tuberculous abscess of the psoas major concurrent with other types of EPTB was 70.77%.

The association network diagram of EPTB diseases concurrent with other types of EPTB shows that almost all the EPTB diseases may be concurrent with other types of EPTB. Tuberculous pleurisy, tuberculous lymphadenitis of the neck, and lumbar vertebra TB ranked among the top three kinds of EPTB concurrent with a number of other types of EPTB disease. Tuberculous pleurisy is the most common EPTB [53]. It is believed that delayed hypersensitivity plays a large role in the pathogenesis of tuberculous pleural effusion [54]. Fluid that enters the pleural space can originate in the pleural capillaries, the interstitial spaces of the lung, the intrathoracic lymphatics, the intrathoracic blood vessels, the peritoneal cavity [55], or the pericardial cavity [56]. The TB bacillus invades the pleural space after the rupture of a pulmonary caseous focus (Ghon focus) in the subpleural region, by contiguity of the pulmonary lesion, by rupture of a mediastinal lymph node, or via hematogenous dissemination [57–59]. Tuberculous pleurisy may cause other types of EPTB via lymphatic or hematogenous dissemination. Pleural lesions can affect not only the abdominal cavity and the subdiaphragmatic organs such as the liver [60] but also the chest wall and even ribs or sternum. Therefore, tuberculous pleurisy is prone to concurrence with other types of EPTB.

Peripheral lymph node TB is one of the most common manifestations of EPTB and one of the most common diseases affecting peripheral lymph nodes [61–63]. The pathogenesis of peripheral mycobacterial lymphadenitis is incompletely understood. Most cases in adults may be the result of the reactivation of lymph node disease sown during lymphatic transmission from a primary respiratory infection [63, 64]. Tuberculous lymphadenitis of the neck may be spread not only by the lymphatic route but also by the hematogenous route from a focus in the lung, genitourinary tract, the skeletal system, or elsewhere [64]. Tuberculous lymphadenitis of the neck may also affect the surrounding skin and soft tissue and can also affect most organs in the head and neck region, such as the lymph nodes, larynx, middle ear, oral cavity, and pharynx [65]. Tuberculous lymphadenitis of the neck may invade the lymphatic system (known as lymphatic metastasis), causing lymph node TB in other parts and distant EPTB through hematogenous metastasis [64]. For the above reasons, tuberculous lymphadenitis of the neck is also more likely to be concurrent with other EPTB.

The proportion of lumbar TB reported in the literature ranges from 22.8% to 45% among all the patients with spinal TB [66, 67]. Lumbar TB concurrent with other types of EPTB is related to the following factors. Firstly, Mtb spreads to the lumbar spine through the blood system, causing lumbar vertebra TB. This disease is not only transmitted through arteries [68], but some investigators favor the possibility that spread of lumbar vertebra TB occurs via Batson's valveless venous vertebral plexus [69, 70]. Secondly, lumbar vertebra TB may originate from adjacent vertebrae bodies and directly invade the lower thoracic vertebrae. Lumbar vertebra TB can cause paraspinal abscess, sacral vertebra TB [71], abdominal cavity viscera TB, and surrounding soft tissue TB. Finally, lumbar vertebra TB may result in the formation of cold abscesses, which release bacteria into the blood that spread via the hematogenous route to remote organs or tissues, leading to distant EPTB [72, 73].

Our study found that about 13% EPTB patients had at least two EPTB lesions. The most common types of EPTB concurrent with other types of EPTB were tuberculous peritonitis concurrent with tuberculous pleurisy (1.64%), tuberculous pericarditis concurrent with tuberculous pleurisy (1.48%), and thoracic vertebra TB concurrent with lumbar TB (0.70%). Abdominal TB may occur directly or indirectly via spread from the primary lesion which generally affects the following organs: lymph nodes, peritoneum, ileocecal junction, colon, liver, spleen, and adrenal glands; solid viscera are affected to a greater extent than the gastrointestinal tract [74]. Tuberculous peritonitis concurrent with tuberculous pleurisy may be caused by lymphatic spread or hematogenous dissemination due to low immunity. Pericardial involvement is caused by the retrograde lymphatic spread of Mtb from peritracheal, peribronchial, or mediastinal lymph nodes or from the hematogenous spread of primary TB infection [75]. The diffusion of Mtb to the pericardium occurs mainly through the rupture of adjacent mediastinal lymph nodes [76]. Tuberculous pericarditis develops secondary to contiguous spread from the mediastinal nodes, lungs, spine, or sternum or during miliary dissemination [77]. The mediastinal pleura and pericardium are adjacent to one another. Tuberculous pericarditis can directly invade the mediastinal pleura, resulting in tuberculous pleurisy. Moreover, it may spread through the blood when the patient's immunity is low, resulting in their concurrence. Approximately 1%–3% of the patients with TB involve infection of the skeletal system, and the spine is the most common target of osteoarticular TB, especially the thoracolumbar spine [78]. There are two possible reasons for thoracic TB concurrence with lumbar TB: firstly, spinal TB occurs through hematogenous dissemination, which can be transmitted from the lungs or via activation of a dormant infection in the bone or joint post trauma [79]. Secondly, the thoracic vertebrae and lumbar vertebrae are adjacent to one another and can be directly invaded [80].

The association rules (confidence and lift) were different between concurrent EPTB. Our study found that sacral TB with concurrent lumbar vertebra TB accounted for 68.56% of sacral TB cases. The confidence value for ureteric TB concurrent with renal TB cases was the second highest (50.52%). Sacral involvement is usually secondary to the lumbar spine and is mainly caused by the direct spread of infection [81]. Urogenital TB accounts for 30%–40% of all extrapulmonary cases [82]. Renal lesions, initially bilateral, cortical, glomerular, and pericapillary, are typically hematogenously spread, accompanied by other hematogenous lesions from other organs aside from the prostate and urogenital system [83]. Starting with a pulmonary focus, 2%–20% of patients developed urogenital TB through hematogenous spread to the kidney, prostate, and epididymis. The spread continued through the descending collection system to the ureter, bladder, and urethra and finally through the ejaculatory duct to the genital organs [84]. Ureteral TB and vesical TB are secondary to downward infection through the collection system. In ureteral TB, multiple strictures develop throughout the ureter; these are mainly anatomical strictures such as vesicoureteral junctions, followed by ureterorenal junctions and strictures at the midureter [85, 86].

### 4.1. Strengths and Limitations

This study had several strengths, including its large-scale multicenter representative sample, its detailed analysis of diagnostic types of concurrent EPTB, and its novel determinations of confidence/lift values of concurrent EPTB. However, the study also had several limitations. First, it may have been subject to Berkson's bias since the study population was all hospitalized TB patients of which concurrent EPTB cases had a high likelihood of being hospitalized, causing the subsequent overestimation of their proportions within the general population. Secondly, most hospitals in our study were TB-specialized hospitals; thus, our findings may not represent the general TB patient population or apply to settings elsewhere in the country. Thirdly, the analysis did not consider low-frequency disease complications and comorbidities in China (e.g., HIV) and other factors such as initial treatment or retreatment, income, and smoking. We did not collect the information of immunocompromised conditions. Fourthly, we could not determine the order of concurrent EPTB for the observational study.

## 5. Conclusions

In conclusion, the present study revealed the association network occurrence of concurrent EPTB types and analyzed the association rules among EPTB for the first time in a large sample population. It was found that almost all the EPTB diseases may be concurrent with other types of EPTB. The number of cases of tuberculous peritonitis concurrent with tuberculous pleurisy, tuberculous pericarditis concurrent with tuberculous pleurisy, and thoracic vertebra TB concurrent with lumbar TB was greater than other types of EPTB. Clinicians should be alert to the incidence of concurrent EPTB and that these patients require administration of customized treatment regimens in order to achieve the best outcomes.

## Figures and Tables

**Table 1:** The characteristics of EPTB inpatients.

Variables	*N* (75,993)	Proportion (%)
*Gender*		
Female	32,738	43.08
Male	43,255	56.92

*Age group*		
15–24 years	15,822	20.82
25–34 years	15,763	20.74
35–44 years	11,326	14.90
45–54 years	11,517	15.16
55–64 years	10,111	13.31
≥ 65 years	11,454	15.07

*EPTB types*		
Exclusively EPTB	66,249	87.18
Concurrent EPTB	9744	12.82

*Note:* Exclusively EPTB: only one EPTB lesion; concurrent EPTB: at least two EPTB lesions.

**Table 2:** Association between gender, age group, and concurrent EPTB in multivariable analysis.

Variables	No. of concurrent EPTB (%)	aOR (95% CI)	*p*
*Gender*			
Female	4466 (13.6)	1.129 (1.081–1.178)	< 0.001
Male	5278 (12.2)	Reference	

*Age group (years)*			
15–24	2184 (13.8)	1.319 (1.225–1.421)	< 0.001
25–34	2188 (13.9)	1.313 (1.219–1.414)	< 0.001
35–44	1527 (13.5)	1.269 (1.172–1.375)	< 0.001
45–54	1424 (12.4)	1.154 (1.064–1.251)	0.001
55–64	1181 (11.7)	1.086 (0.998–1.182)	0.057
≥ 65	1240 (10.8)	Reference	
Trend test	—	0.945 (0.933–0.957)	< 0.001

**Table 3:** The proportion of concurrent EPTB > 50% in EPTB disease (≥ 100 cases).

EPTB types	*n*	Concurrent EPTB proportion (%)
Ureteric tuberculosis	194	78.87
Sacral tuberculosis	229	78.60
Tuberculous abscess of psoas major	349	70.77
Vesical tuberculosis	165	69.70
Hepatic tuberculosis	148	63.51
Tuberculosis of abdominal lymph nodes	211	58.77
Tuberculosis of mediastinal lymph nodes	687	55.75
Tuberculous pericarditis	2409	54.67
Oviduct tuberculosis	167	52.69
Brain tuberculoma	104	50.96

*Note: n*-the case of EPTB disease. Concurrent EPTB proportion − (concurrent EPTB)/(concurrent EPTB + exclusively EPTB).

**Table 4:** The concurrent types (≥ 40) in EPTB disease (≥ 100 cases).

EPTB types	*n* (%)	No. of concurrent other types of EPTB (%)
Tuberculous pleurisy	35,316 (46.47)	66 (82.50%)
Tuberculous lymphadenitis of the neck	7974 (10.49)	64 (80.00%)
Lumbar vertebra tuberculosis	4504 (5.93)	59 (73.75%)
Tuberculous meningitis	5485 (7.22)	53 (66.25%)
Thoracic vertebra tuberculosis	2786 (3.67)	51 (63.75%)
Tuberculous peritonitis	4157 (5.47)	48 (60.00%)
Chest wall tuberculosis	2343 (3.08)	48 (60.00%)
Renal tuberculosis	1579 (2.08)	48 (60.00%)
Tuberculous empyema	3475 (4.57)	46 (57.50%)
Cutaneous tuberculosis	467 (0.61)	44 (55.00%)
Tuberculosis of mediastinal lymph nodes	687 (0.90)	43 (53.75%)
Intestinal tuberculosis	1278 (1.68)	41 (51.25%)
Tuberculous pericarditis	2409 (3.17)	40 (50.00%)
Knee joint tuberculosis	667 (0.88)	40 (50.00%)

*Note:* Concurrent types—one type of EPTB concurrent with the number of other types of EPTB. *n*% = *n*/75993 ∗ 100%.

**Table 5:** The cases of concurrent EPTB types (cases ≥ 100).

Concurrent EPTB types	*n*	Proportion (%)
Tuberculous peritonitis concurrent with tuberculous pleurisy	1249	1.64
Tuberculous pericarditis concurrent with tuberculous pleurisy	1124	1.48
Thoracic vertebra tuberculosis concurrent with lumbar vertebra tuberculosis	533	0.70
Tuberculous meningitis concurrent with tuberculous pleurisy	444	0.58
Tuberculous lymphadenitis of neck concurrent with tuberculous pleurisy	406	0.53
Bronchial tuberculosis concurrent with tuberculous pleurisy	384	0.51
Intestinal tuberculosis concurrent with tuberculous peritonitis	314	0.41
Chest wall tuberculosis concurrent with tuberculous pleurisy	279	0.37
Pelvic tuberculosis concurrent with tuberculous peritonitis	276	0.36
Thoracic vertebra tuberculosis concurrent with tuberculous pleurisy	274	0.36
Chest wall tuberculosis concurrent with tuberculous empyema	253	0.33
Tuberculous empyema concurrent with tuberculous pleurisy	207	0.27
Lumbar vertebra tuberculosis concurrent with tuberculous pleurisy	184	0.24
Tuberculosis of axillary lymph nodes concurrent with tuberculous lymphadenitis of neck	175	0.23
Bronchial tuberculosis concurrent with tuberculous lymphadenitis of neck	172	0.23
Sacral tuberculosis concurrent with lumbar vertebra tuberculosis	157	0.21
Lumbar vertebra tuberculosis concurrent with tuberculous abscess of psoas major	153	0.20
Tuberculous peritonitis concurrent with tuberculous pericarditis	145	0.19
Tuberculosis of mediastinal lymph nodes concurrent with tuberculous lymphadenitis of neck	142	0.19
Intestinal tuberculosis concurrent with tuberculous pleurisy	136	0.18
Lumbar vertebra tuberculosis concurrent with tuberculous meningitis	136	0.18
Pelvic tuberculosis concurrent with tuberculous meningitis	115	0.15

*Note:* Proportion = *n* ∗ 100%/75,993.

**Table 6:** The association rules of concurrent EPTB (mininstances = 100, minconfidence = 20%, and lift ≥ 1).

Consequent	Antecedent	ID	Instances	Support (%)	Confidence (%)	Lift
Lumbar vertebra tuberculosis	Sacral tuberculosis	1	229	0.30	68.56	11.57⁣^∗^
Renal tuberculosis	Ureteric tuberculosis	2	194	0.26	50.52	24.31⁣^∗^
Tuberculous pleurisy	Tuberculous pericarditis	3	2409	3.17	46.66	1.01
Renal tuberculosis	Vesical tuberculosis	4	165	0.22	45.45	21.88⁣^∗^
Lumbar vertebra tuberculosis	Tuberculous abscess of psoas major	5	349	0.46	43.84	7.40⁣^∗^
Ureteric tuberculosis	Vesical tuberculosis	6	165	0.22	42.42	166.18⁣^∗^
Vesical tuberculosis	Ureteric tuberculosis	7	194	0.26	36.08	166.18⁣^∗^
Tuberculous meningitis	Brain tuberculoma	8	104	0.14	30.77	4.26⁣^∗^
Tuberculous peritonitis	Pelvic tuberculosis	9	1039	1.37	26.56	4.86⁣^∗^
Tuberculous lymphadenitis of neck	Inguinal lymph node tuberculosis	10	245	0.32	26.53	2.53
Pelvic tuberculosis	Oviduct tuberculosis	11	167	0.22	26.34	19.27⁣^∗^
Tuberculous lymphadenitis of neck	Tuberculosis of axillary lymph nodes	12	703	0.93	24.89	2.37
Tuberculous peritonitis	Intestinal tuberculosis	13	1278	1.68	24.57	4.49⁣^∗^
Pelvic tuberculosis	Endometrial tuberculosis	14	185	0.24	23.24	17.00⁣^∗^
Tuberculosis of mediastinal lymph nodes	Tuberculosis of hilar lymph nodes	15	205	0.27	22.44	24.82⁣^∗^
Tuberculous lymphadenitis of neck	Tuberculosis of mediastinal lymph nodes	16	687	0.90	20.67	1.97
Tuberculous peritonitis	Hepatic tuberculosis	17	148	0.19	20.27	3.71⁣^∗^

*Note:* ID displays the sequence of the association rules. Instances display the cases of EPTB lesion. Mininstances: the lowest of instance. Minconfidence: the lowest of confidence.

⁣^∗^Lift ≥ 3.

## Data Availability

The datasets used or analyzed during the current study are available from the corresponding authors on reasonable request.
